# Associations of life’s essential 8 with MAFLD and liver fibrosis among US adults: a nationwide cross-section study

**DOI:** 10.3389/fnut.2024.1403720

**Published:** 2024-06-12

**Authors:** Depeng Liang, Jie Zhang, Lu Li, Yonggang Li, Lidong Xu, Huili Wu

**Affiliations:** ^1^Department of Gastroenterology, Zhengzhou Central Hospital Affiliated to Zhengzhou University, Zhengzhou, China; ^2^Henan Provincial Medicine Key Laboratory of Colorectal Cancer Diagnosis and Treatment, Zhengzhou, China; ^3^Zhengzhou Key Laboratory of Colorectal Cancer Diagnosis, Treatment and Research, Zhengzhou, China

**Keywords:** NHANES, life’s essential 8, MAFLD, liver fibrosis, cross section study

## Abstract

**Background:**

Life’s essential’ 8 (LE8) is a newly updated cardiovascular health (CVH) metrics from the American Heart Association, with close relevance to metabolism. Our objective is to explore the association between LE8 scores and incidence of metabolic dysfunction-associated fatty liver disease (MAFLD) and advanced liver fibrosis in American adults.

**Methods:**

This population-based cross-sectional study utilized data from the National Health and Nutrition Examination Survey (NHANES) conducted between 2005 and 2018, encompassing adults aged 20 years or older. Validated non-invasive scoring systems were employed to define liver steatosis and advanced liver fibrosis. Multivariable logistic regression and smooth curve fitting techniques were applied to evaluate the associations. All analyses were adjusted for the survey’ complex design parameters and accounted for sample weights.

**Results:**

A total of 11,820 participants were included. A higher LE8 score was found to be inversely associated with the incidence of MAFLD and advanced liver fibrosis, with odds ratios (OR) of 0.64 (95% CI: 0.57–0.71) for MAFLD and 0.75 (95% CI: 0.61–0.92) for advanced liver fibrosis per 1 standard deviation (SD) increase in LE8 score. Similar patterns were found in the relationship between health behaviors/factors score and incidence of MAFLD and advanced liver fibrosis. In subgroup analyses, the interaction test showed that age, education level, marital status, CVD, hypertension and diabetes had a significant impact on the association between LE8 score and MAFLD (all *P* for interaction < 0.05). Among male, elderly, wealthy, other race, CVD, diabetes and depression participants, the correlation between LE8 score and advanced liver fibrosis was not statistically significant (*P* > 0.05). Younger participants exhibited a more pronounced negative association between the CVH metric and both MAFLD and advanced life fibrosis.

**Conclusion:**

LE8 and its subscales score were inversely associated with the presence of MAFLD and advanced liver fibrosis in non-linear patterns. Optimal LE8 score may significantly reduce the risk of liver steatosis and fibrosis.

## 1 Introduction

As we all know, Non-alcoholic fatty liver disease (NAFLD) is a common chronic non-communicable disease because of its increasing prevalence, affecting up to nearly 30% of the general adult population, which brings a substantial health and economic burden to the society (1–3). Previous prior studies have shown that NAFLD is closely associated with metabolic syndrome, including increased risks of diabetes, obesity, kidney disease, and cardiovascular disease, as well as an increased risk of cirrhosis and other complications. Both obesity and aging are associated with increased prevalence of NAFLD and liver fibrosis (4). In an Italian cohort study, a higher stage of fibrosis was observed in elderly subjects, along with metabolic disorders. For the elderly, these changes make them more susceptible to a variety of non-communicable diseases, including cancer, cardiovascular disease, hypertension, diabetes, cerebrovascular disease (5). However, NAFLD is an exclusive diagnosis that excludes viral hepatitis, alcoholic hepatitis, drug-induced hepatitis, and other diseases and carries the suspicion of stigma. It itself ignores the correlation with metabolism and intra-patient heterogeneity. In 2020, experts aiming to better understand the relationship between fatty liver and metabolism in the field of liver disease from around the world issued an initiative proposing to rename NAFLD to metabolic dysfunction-associated fatty liver disease (MAFLD) and to switch to positive diagnostic criteria (6). The MAFLD diagnostic criteria are more advantageous in diagnosing metabolic dysfunction, extrahepatic illnesses, and high-risk liver disorders, as well as in predicting liver fibrosis (7). Therefore, MAFLD can accurately reflect metabolic disorders as the underlying mechanism of the disease, which is more in line with the diagnostic logic of the disease, and stratified management of patients is beneficial.

In 2010, the American Heart Association (AHA) proposed an indicator to assess the general state of health in the US population, known as Life’s Simple 7 (LS7) or cardiovascular health (CVH) metrics, in order to evaluate the status of cardiovascular disease in Americans and further promote health awareness among the general population (8). Subsequently, many studies have found that having a higher CVH decreases the risk of developing cardiovascular diseases, cancers, dementia, advanced renal disease, and chronic obstructive pulmonary disease (9–11). It has been demonstrated that the evaluation of CVH status is a beneficial instrument for monitoring individual as well as community health throughout the entire life cycle. However, the original CVH metrics ignored responsiveness to individual variation and changes. Therefore, AHA would update the evaluation tool for CVH quantification, namely “Life’s Essential 8” (LE8) in 2022 (12). Several studies have indicated that in adults aged 20 and above in the United States, higher Cardiovascular Health (CVH) measured by the LE8 score is inversely associated with various non-communicable diseases (11). Participants with high LE8 score exhibit more ideal cardiovascular health (13), significantly reducing the risks of new-onset NAFLD, depression and anxiety events, biological aging, extending life expectancy (14–16). Sleep health is included as a new component of LE8 and the measurement method for other components has a more reasonable definition and quantification. The revised LE8 reacts well to these factors and is more cognizant of individual differences. People are increasingly realizing the crucial importance of social determinants of health for CVH (17).

The risk factors for CVD and NAFLD are closely correlated (18, 19). Promoting CVH may be a useful management and preventative approach for reducing the impact of NAFLD. Studies have demonstrated that adherence to the ideal cardiovascular health (CVH) index, as defined by the Life’s Simple 7 (LS7) metrics, is associated with a significant reduction in the risk of NAFLD and liver fibrosis (20). Wang’s study suggests that higher levels of LE8 are associated with a lower incidence of NAFLD, but the score for healthy behaviors is not associated with NAFLD and does not reveal the relationship between LE8 and liver fibrosis (21). It is currently unclear what the relationship is between MAFLD, advanced liver fibrosis, and LE8 in the adult population of the United States.

Hence, the primary aim of this study is to investigate a comprehensive preventive strategy aimed at reducing the incidence of metabolic-associated fatty liver disease (MAFLD) by examining the association between MAFLD, liver fibrosis, and updated LE8 score among adult Americans. Developing effective prevention and standardized management strategies for MAFLD to reduce the socio-economic burden in liver diseases.

## 2 Materials and methods

### 2.1 Design and study population

The NHANES program is a cross-sectional, continuous wellness survey. The purpose of using complex, multi-stage, and probabilistic clustering design in research is to represent the entire non-institutionalized civilian population of the United States (22). The research strategy was approved by the National Center for Health Statistics Ethics Review Committee, and informed consent was acquired via documentation from each participant.^1^ This study follows the reporting guidelines of the Epidemiological Observational Study Guidelines (STROBE) (23).

This study analyzed data from six NHANES survey cycles from 2005 to 2018, with a total of 70,190 participants. We have established exclusion criteria: (1) Excluding participants under the age of 20 and pregnant women (*n* = 30,441); (2) Missing LE8 components (*n* = 12,763); missing MAFLD information (*n* = 1,421); (3) Missing NFS information (*n* = 57), triglyceride (*n* = 107), glycated hemoglobin (*n* = 19), low-density lipoprotein (*n* = 199), education level (*n* = 8), marital status (*n* = 1), and uric acid (*n* = 1) were excluded. In addition, 563 individuals had the sample weight values of 0 or were missing sample weights, therefore their data will not be considered in the weighted analysis. Ultimately, there were 11,820 adult participants in the research (Supplementary Figure 1).

### 2.2 Definitions of life’s essential 8

According to the update of AHA’s cardiovascular health structure, four health behaviors (diet, physical activity, nicotine exposure, and sleep health) and four health factors (body mass index, blood lipids, blood sugar, and blood pressure) are included in the LE8 scoring procedures (12, 24). The detailed algorithm for each part of LE8 scoring has already been uploaded and is included in Supplementary Table 2. The individuals’ two 24-h meal recalls from the NHANES database were used to measure dietary conditions, which were then evaluated using the Healthy Eating Index (HEI) 2015 (25). Physical activity, nicotine exposure, sleep health data, diabetes history and drug history were collected through self-reported questionnaires. Plasma total cholesterol and HDL cholesterol, blood glucose, and glycosylated hemoglobin were measured using blood samples collected on an empty stomach. According to the AHA guidelines, each of the CVH metrics has a rating range of 0–100 points, and the total score of LE8 is the unweighted mean of the 8 metrics (17). Participants with a LE8 score of 80–100 is considered as the high CVH; 50–79 is considered the moderate CVH; 0–49 is considered as the low CVH (12). We quantified and classified health behaviors/factors scores based on consistent criteria and cutoff points, and subsequently examined the correlation between MAFLD, liver fibrosis, and these scores.

### 2.3 Definitions of MAFLD and advanced liver fibrosis

Hepatic steatosis in our study was determined by utilizing the US Fatty Liver Index (USFLI), which is calculated based on a previously established formula derived from the NHANES. A USFLI score exceeding 30 was used as the threshold for defining hepatic steatosis, with an area under the receiver operating characteristic curve (AUROC) of 0.80, demonstrating a sensitivity of 62% and a specificity of 88% (26). The positive diagnosis of MAFLD is based on the USFLI to define liver steatosis as a substitute for liver biopsy, as evidence of fat accumulation in the liver (liver steatosis), and one of the following three criteria (6, 27): overweight/obesity (body weight refers to [BMI] ≥ 25 kg/m^2^); Self-reported history of diabetes, using insulin to reduce blood sugar, or glycosylated hemoglobin (HbA1c) ≥ 6.5%; Or lean or normal weight with at least two or more metabolic risk disorders, including: 1. White men and women with a waist circumference of ≥ 102/88 cm; 2. blood pressure ≥ 130/85 mmHg or specific medication treatment; 3. Plasma triglycerides ≥ 150 mg/dl or specific drug treatment; 4. Male plasma high-density lipoprotein cholesterol (HDL) < 40 mg/dl, female plasma high-density lipoprotein cholesterol (HDL) < 50 mg/dl, or specific drug treatment; 5. Pre-diabetes (fasting blood glucose 100–125 mg/dl, or glycosylated hemoglobin 5.7–6.4%; 6.HOMA-IR score ≥ 2.5); it is worth noting that due to the survey design, C-reactive protein cannot be obtained in NHANES 2011–2014, so it is excluded from this standard. Comprehensive details regarding the laboratory procedures are available at: https://wwwn.cdc.gov/Nchs/Nhanes.

The NAFLD fibrosis score (NFS) system proposed by Angulo et al. (28) showed high accuracy in diagnosing progressive fibrosis in MAFLD, so this study used NFS as an evaluation indicator for liver fibrosis. Early studies indicate that liver fibrosis defined by The NAFLD fibrosis score (NFS) predicts increased mortality (5). Liver fibrosis appears to be a major determinant of disease progression, with individuals exhibiting higher degrees of liver fibrosis more likely to experience poorer long-term prognosis (29). Age is the primary factor leading to the rate of liver fibrosis and its adverse health outcomes. McPherson et al. suggest that when assessing fibrosis risk in individuals aged 65 and older, the critical threshold for NFS should be set at 0.676. This enhances the specificity for detecting high-risk advanced fibrosis, effectively controls false-positive rates, and mitigates the adverse increase in false-negative rates (30, 31). It is key to note that the definitions of both MAFLD and liver fibrosis are based on non-invasive scores.

NFS = −1.675 + 0.037 × age + 0.094 × BMI + 1.13 × impaired fasting glycemia or diabetes (yes = 1, no = 0) + 0.99 × AST/ALT ratio − 0.013 × platelet − 0.66 × albumin.

### 2.4 Ascertainment of other covariates

The interview defined age, gender (male, female), and race/ethnicity (Mexican American, Other Hispanic, Non-Hispanic White, Non-Hispanic Black, and Other Race). Education level is divided into high school graduates or below, partial university graduates, and university graduates or above. Marital status was categorized as Married/Living with a Partner, Divorced/Separated/Widowed, and Never Married. The poverty ratio was categorized as the ratio of monthly family income to poverty levels and categorized into 3 groups: < 1.3 (low income), 1.3–3.5 (middle income), > 3.5 (high income), and missing. A history of CVD is a previous diagnosis of heart failure, coronary heart disease, angina, heart attack, or stroke. Body measurements, including height and weight, were collected during visits to a mobile examination center (MEC), and the body mass index (BMI) was calculated using the formula weight/height^2^; The alcohol consumption is the average of the past two 24-h alcohol consumptions in NHANES dietary data. The classification of smoking is as follows: current smoking (smoking ≥ 100 cigarettes and current smokers); previous smoking (smoking ≥ 100 cigarettes but not currently smoking); never smoking (never smoking or smoking ≤ 100 cigarettes); A cancer history was established based on the self-report of the NHANES medical condition questionnaire. Depression (PHQ-9) is a scale used for evaluating the severity of depression in individuals with a total score of 0–27. A PHQ-9 score of ≥ 10 points is used to diagnose depression (32). Hypertension and diabetes were diagnosed through measurement indicators, previous drug usage, and self-reported questionnaire. Cancer was determined through self-reported questionnaire.

### 2.5 Statistical analysis

All analyses in this research were conducted using R software (v.4.2.3; R statistical computing base, Vienna, Austria), SPSS (v.25.0; IBM Corporation, Armonk, NY, USA), and Empower software^2^ (X&Y Solutions, Inc., Boston, MA) running on R software. Empower software and R were both available for open access. For all analyses, the statistical significance level was determined as bilateral *p* < 0.05, and the 95% confidence interval was calculated in this study. The complicated multi-stage stratified sampling strategy of the NHANES is illustrated statistically through the use of weights.

We selected WTSAF2YR-Fasting Subsample 2 Year MEC Weight to adjust for bias estimates caused by complex multi-stage sampling, and adjusted the sample weights in accordance with the weighting standards provided by NHANES (33): MTS14YR = 1/7*WTSAF2YR (WTSAF2YR is the 2-year subsample weight in each survey period; MTS14YR is the sample weight determined through combining the seven survey periods).

In this study, the quantitative variables in the characteristics of the participants were expressed as mean ± standard error, and the qualitative variables were expressed as percentage or frequency. To exclude the problem of multicollinearity, we used the multicollinearity test, when a variance inflation factor (VIF) greater than 5 was considered to have a collinearity problem (Supplementary Table 1). Weighted linear regression and weighted chi square tests were used to assess the statistical significance of the quantitative and qualitative variables. Differences in the prevalence rates of MAFLD and NFS categories and other categorical variables and their 95% confidence intervals (CI) were calculated and used to assess important practical differences in the magnitude of association, i.e., effect size (ES) (5). Differences between continuous variables were calculated using Cohen’s d difference between means and their ES using confidence intervals around them. Evaluate the correlation between LE8 and each part and MAFLD or advanced liver fibrosis in weighted multivariate logistic regression analysis and establish an adjusted model based on the included covariates. Model 1 did not adjust the variables. Model 2 was adjusted based on age, gender, race, education level, marital status, and PIR. Model 3 is a fully adjusted model with further adjustments for additionally adjusted BMI (< 25, 25–30, ≥ 30), GGT, HDL, TG, ALT, AST, ALP, alcohol, albumin, smoking, hypertension, CVD, diamonds, compression, and cancer. Due to the use of the LE8 score to define and quantify cardiovascular health, when evaluating the correlation between LE8 and MAFLD. To test the robustness of our results, Model 4 did not include individuals who had self-reported history of cardiovascular disorders, such as coronary heart disease, angina, heart attacks, or strokes. In multivariate logistic regression, LE8/CVH was divided into three groups: low level (0–49, *n* = 1,410), medium level (50–79, *n* = 7,941), and high level (80–100, *n* = 2,469). When low CVH is employed as the control group, we calculated the z-score of LE8 and reported the odds ratio (OR) of MAFLD and advanced liver fibrosis for each standard deviation (SD) increase in LE8. Smooth curve fitting was used to observe the dose-response relationship between LE8 and MAFLD, advanced liver fibrosis. In addition, a stratified multivariable logistic regression model was used to carry out a subgroup analysis of the correlation between LE8 and MAFLD and advanced liver fibrosis. The significance of the interaction was estimated using the *P*-value of the production items between the stratified components and LE8.

## 3 Results

### 3.1 Baseline characteristics

The baseline characteristics of 11,820 participants with available data for analysis are shown in Table 1, representing approximately 172.1 million American adults aged ≥ 20 years. Low, moderate, and high CVHs were used to categorize the research population’s baseline characteristics. Among the subjects, the weighted average age of the participants was 47.76 ± 0.28 years old, 52.22% were female. There were 1,410 cases (11.93%), 7,941 cases (67.18%), and 2,469 cases (20.89%) with low, moderate, and high CVH, respectively. Almost all characteristics showed significant differences among the various CVH metric groups (*p* < 0.05). The mean age exhibited a decreasing trend across each increasing LE8 score groups during the transition from the low CVH group to the moderate CVH group (ES: 0.19, 95% CI: 0.13, 0.25) and from the moderate CVH group to the high CVH group (ES: 0.26, 95% CI: 0.22, 0.31). The high CVH group had a lower burden of hypertension, CVD, diabetes, cancer and depression, and there is a lower blood lipid level, such as Triglyceride levels (ES: 1.03, 95% CI: 0.97, 1.10), LDL (ES: 0.53, 95% CI: 0.46, 0.60), as well as lower uric acid levels (ES: 0.56, 95% CI: 0.50, 0.63), HBA1C levels (ES: 0.90, 95% CI: 0.83, 0.97) and BMI (ES: 1.29, 95% CI: 1.22, 1.37) in low vs. high LE8 score groups. The high CVH group’s participants were higher education levels, wealthier, less divorced/separated/ widowed, and fewer smokers. As the LE8 score increases, liver function indicators exhibit a consistent trend of decrease. In particular, the average liver enzymes in the high CVH score group were lower than those in the low CVH and moderate CVH groups (ES: 0.57, 95% CI: 0.50, 0.63 and ES: 0.23, 95% CI: 0.19, 0.28 for ALP levels; ES: 0.23, 95% CI: 0.17, 0.30 and ES: 0.19, 95% CI: 0.15, 0.24 for ALT levels, and ES: 0.52, 95% CI: 0.45, 0.58 and ES: 0.22, 95% CI: 0.17, 0.26 for GGT levels, respectively). No meaningful difference was observed for AST and alcohol across groups. What’s more, we found that individuals with low CVH were more likely to get MAFLD (low CVH: 67.09%, moderate CVH: 35.71%, high CVH: 5.52%, *p* < 0.05) and advanced liver fibrosis (low CVH: 17.01%, moderate CVH: 6.07%, high CVH: 1.04%, *p* < 0.05).

**TABLE 1:** General characteristics of participants by cardiovascular health [Weighted (11,820)].

Characteristic	LE8 score^†^			Effect size^‡^		
	Low CVH (*N* = 1,410)	Moderate CVH (*N* = 7,941)	High CVH (*N* = 2,469)	Low vs. moderate	Low vs. high	Moderate vs. high
Age, mean (SE)	53.89 (0.56)	48.92 (0.30)	42.14 (0.47)	0.19 (0.13, 0.25)	0.52 (0.45, 0.59)	0.26 (0.22, 0.31)
Alcohol, mean (SE)	6.06 (0.59)	9.27 (0.42)	9.59 (0.51)	−0.09 (−0.15, −0.03)	−0.15 (−0.21, −0.08)	−0.01 (−0.05, 0.04)
BMI, mean (SE)	35.00 (0.30)	29.64 (0.09)	24.55 (0.11)	0.62 (0.57, 0.68)	1.29 (1.22, 1.37)	0.68 (0.63, 0.73)
HDL, mean (SE),	47.87 (0.44)	53.24 (0.22)	61.73 (0.52)	−0.28 (−0.34, −0.22)	−0.61 (−0.67, 0.54)	−0.40 (−0.45, −0.35)
Triglyceride, mean (SE)	159.42 (2.71)	121.94 (1.00)	82.01 (1.08)	0.41 (0.35, 0.47)	1.03 (0.97, 1.10)	0.49 (0.44, 0.53)
LDL, mean (SE)	126.81 (1.58)	116.71 (0.57)	101.84 (0.77)	0.19 (0.14, 0.25)	0.53 (0.46, 0.60)	0.31 (0.26, 0.35)
HBA1C, mean (SE)	6.32 (0.05)	5.58 (0.01)	5.24 (0.01)	0.67 (0.62, 0.73)	0.90 (0.83, 0.97)	0.42 (0.37, 0.46)
Albumin, mean (SE)	40.93 (0.14)	42.29 (0.06)	43.31 (0.08)	−0.26 (−0.31, −0.20)	−0.53 (−0.60, −0.46)	−0.20 (−0.25, −0.16)
Total bilirubin, mean (SE)	10.49 (0.17)	11.88 (0.10)	12.93 (0.16)	−0.16 (−0.22, −0.11)	−0.33 (−0.39, −0.26)	−0.12 (−0.17, −0.08)
Uric acid, mean (SE)	352.87 (3.54)	332.19 (1.22)	292.52 (1.80)	0.18 (0.13, 0.24)	0.56 (0.50, 0.63)	0.38 (0.33, 0.43)
ALT, mean (SE)	26.36 (0.63)	25.83 (0.25)	21.78 (0.34)	0.02 (−0.03, 0.08)	0.23 (0.17, 0.30)	0.19 (0.15, 0.24)
AST, mean (SE)	24.69 (0.46)	25.27 (0.23)	24.00 (0.34)	−0.03 (−0.09, 0.03)	0.04 (−0.02, 0.11)	0.06 (0.02, 0.11)
ALP, mean (SE)	76.68 (0.92)	69.21 (0.44)	60.83 (0.47)	0.19 (0.14, 0.25)	0.57 (0.50, 0.63)	0.23 (0.19, 0.28)
GGT, mean (SE)	36.57 (1.32)	28.71 (0.57)	18.70 (0.44)	0.16 (0.10, 0.21)	0.52 (0.45, 0.58)	0.22 (0.17, 0.26)
LE8 scores	42.39 (0.22)	66.28 (0.14)	86.83 (0.14)	−2.00 (−2.07, −1.94)	−5.96 (−6.11, −5.81)	−1.80 (−1.85, −1.75)
Health behaviors score	38.08 (0.57)	64.00 (0.29)	84.25 (0.26)	−1.03 (−1.09, −0.97)	−2.80 (−2.89, −2.71)	−0.86 (−0.91, −0.82)
Health factors score	46.70 (0.56)	68.56 (0.25)	89.41 (0.23)	−0.99 (−1.05, −0.93)	−2.74 (−2.82, −2.65)	−1.03 (−1.08, −0.98)
HEI-2015 diet score	20.11 (0.86)	34.06 (0.51)	58.58 (0.86)	−0.32 (−0.38, −0.26)	−0.98 (−1.05, −0.91)	−0.55 (−0.59, −0.50)
Physical activity score	26.41 (1.63)	70.48 (0.72)	93.75 (0.55)	−0.69 (−0.75, −0.63)	−1.57 (−1.65, −1.50)	−0.40 (−0.45, −0.36)
Nicotine exposure score	39.97 (1.63)	68.50 (0.70)	91.85 (0.52)	−0.46 (−0.52, −0.40)	−1.23 (−1.30, −1.16)	−0.42 (−0.46, −0.37)
Sleep health score	65.82 (1.12)	82.96 (0.38)	92.82 (0.35)	−0.49 (−0.54, −0.43)	−0.93 (−1.00, −0.87)	−0.32 (−0.37, −0.28)
Body mass index score	30.73 (1.13)	56.74 (0.45	85.38 (0.54)	−0.64 (−0.70, −0.59)	−1.64 (−1.71, −1.56)	−0.77 (−0.81, −0.72)
Blood lipids score	46.33 (1.11)	63.10 (0.45)	83.43 (0.56)	−0.42 (−0.47, −0.36)	−1.11 (−1.18, −1.04)	−0.54 (−0.59, −0.50)
Blood glucose score	63.15 (1.09)	87.03 (0.33)	98.22 (0.26)	−0.76 (−0.82, −0.70)	−1.31 (−1.38, −1.24)	−0.42 (−0.47, −0.38)
Blood pressure score	46.57 (1.01)	67.38 (0.52)	90.63 (0.45)	−0.46 (−0.52, −0.40)	−1.52 (−1.59, −1.45)	−0.55 (−0.60, −0.51)
**Gender, percent (SE)**						
Male	45.14 (1.75)	51.01 (0.61)	39.98 (1.32)	5.87 (3.05, 8.69)	−5.16 (−8.40, −1.92)	−11.03 (−13.25, −8.81)
Female	54.86 (1.75)	48.99 (0.61)	60.02 (1.32)	−5.87 (−8.69, −3.05)	5.16 (1.92, 8.40)	11.03 (8.81, 13.25)
**Race/ethnicity, percent (SE)**						
Mexican American	7.41 (1.01)	7.91 (0.65)	7.69 (0.74)	0.50 (−0.99, 1.99)	0.28 (−1.44, 2.00)	−0.22 (−1.43, 0.99)
Other Hispanic	5.02 (0.72)	5.29 (0.50)	5.50 (0.63)	0.27 (−0.97, 1.51)	0.48 (−0.97, 1.93)	0.21 (−0.82, 1.24)
Non-Hispanic White	66.02 (2.04)	69.24 (1.39)	70.95 (1.51)	3.22 (0.55, 5.89)	4.93 (1.88, 7.98)	1.71 (−0.35, 3.77)
Non-Hispanic Black	17.05 (1.42)	11.25 (0.74)	6.34 (0.59)	−5.80 (−7.88, −3.72)	−10.71 (−12.90, −8.52)	−4.91 (−6.10, −3.72)
Other Race	4.50 (0.76)	6.32 (0.42)	9.53 (0.89)	1.82 (0.61, 3.03)	5.03 (3.44, 6.62)	3.21 (1.93, 4.49)
**Marital status, percent (SE)**						
Married/living with a partner	59.08 (1.87)	65.20 (0.81)	67.49 (1.41)	6.12 (3.35, 8.89)	8.41 (5.25, 11.57)	2.29 (0.17, 4.41)
Divorced/separated/widowed	26.56 (1.57)	19.31 (0.66)	9.97 (0.68)	−7.25 (−9.71, −4.79)	−16.59 (−19.18, −14.00)	−9.34 (−10.81, −7.87)
Never married	14.37 (1.41)	15.49 (0.66)	22.54 (1.24)	1.12 (−0.88, 3.12)	8.17 (5.71, 10.63)	7.05 (5.22, 8.88)
**Education levels, percent (SE)**						
High school or less	60.86 (2.00)	40.80 (1.08)	20.44 (1.18)	−20.06 (−22.83, −17.29)	−40.42 (−43.42, −37.42)	−20.36 (−22.28, −18.44)
College or associates degree	27.23 (1.53)	33.07 (0.75)	26.99 (1.43)	5.84 (3.30, 8.38)	−0.24 (−3.15, 2.67)	−6.08 (−8.11, −4.05)
College graduate or above	11.91 (1.42)	26.13 (1.07)	52.57 (1.62)	14.22 (12.27, 16.17)	40.66 (38.06, 43.26)	26.44 (24.25, 28.63)
**Poverty ratio, percent (SE)**						
< 1.3	30.30 (1.66)	18.63 (0.80)	13.17 (0.77)	−11.67 (−14.22, −9.12)	−17.13 (−19.87, −14.39)	−5.46 (−7.05, −3.87)
1.3–3.5	41.43 (1.82)	35.80 (0.92)	28.67 (1.41)	−5.63 (−8.41, −2.85)	−12.76 (−15.89, −9.63)	−7.13 (−9.20, −5.06)
≥ 3.5	21.31 (1.86)	39.80 (1.24)	52.37 (1.53)	18.49 (16.10, 20.88)	31.06 (28.15, 33.97)	12.57 (10.33, 14.81)
Missing	6.96 (0.95)	5.77 (0.41)	5.79 (0.66)	−1.19 (−2.61, 0.23)	−1.17 (−2.79, 0.45)	0.02 (−1.03, 1.07)
**Smoking, percent (SE)**						
Never	23.14 (1.65)	51.07 (0.89)	79.12 (1.14)	27.93 (25.47, 30.39)	55.98 (53.26, 58.70)	28.05 (26.11, 29.99)
Former	28.34 (1.61)	28.01 (0.72)	19.13 (1.11)	−0.33 (−2.88, 2.22)	−9.21 (−12.03, −6.39)	−8.88 (−10.72, −7.04)
Current	48.52 (1.88)	20.92 (0.71)	1.75 (0.35)	−27.60 (−30.36, −24.84)	−46.77 (−49.43, −44.11)	−19.17 (−20.20, −18.14)
**Depression, percent (SE)**						
No	80.14 (1.42)	89.85 (0.58)	93.62 (0.61)	9.71 (7.52, 11.90)	13.48 (11.19, 15.77)	3.77 (2.60, 4.94)
Yes	16.40 (1.31)	6.78 (0.38)	2.61 (0.39)	−9.62 (−11.63, −7.61)	−13.79 (−15.82, −11.76)	−4.17 (−5.01, −3.33)
Missing	3.46 (0.60)	3.38 (0.39)	3.78 (0.44)	−0.08 (−1.11, 0.95)	0.32 (−0.89, 1.53)	0.40 (−0.45, 1.25)
**AGE categorical, percent (SE)**						
< 40	16.80 (1.37)	32.34 (0.77)	49.80 (1.59)	15.54 (13.33, 17.75)	33.00 (30.23, 35.77)	17.46 (15.24, 19.68)
≥ 40, < 60	45.97 (1.70)	39.31 (0.71)	33.10 (1.47)	−6.66 (−9.47, −3.85)	−12.87 (−16.07, −9.67)	−6.21 (−8.35, −4.07)
≥60	37.23 (1.94)	28.35 (0.81)	17.10 (1.09)	−8.88 (−11.59, −6.17)	−20.13 (−23.06, −17.20)	−11.25 (−13.04, −9.46)
CVD history, percent (SE)	22.35 (1.55)	8.70 (0.45)	3.17 (0.45)	−13.65 (−15.91, −11.39)	−19.18 (−21.46, −16.89)	−5.53 (−6.46, −4.60)
Hypertension, percent (SE)	70.37 (1.69)	41.97 (0.78)	11.56 (0.77)	−28.4 (−31.02, −25.78)	−58.81 (−61.51, −56.11)	−30.41 (−32.07, −28.75)
Diabetes, percent (SE)	37.03 (1.89)	12.09 (0.45)	1.26 (0.36)	−24.94 (−27.56, −22.32)	−35.77 (−38.33, −33.21)	−10.83 (−11.67, −9.99)
Cancer, percent (SE)	10.24 (0.98)	10.36 (0.46)	7.35 (0.67)	0.12 (−1.60, 1.84)	−2.89 (−4.78, −1.00)	−3.01 (−4.24, −1.78)
MAFLD, percent (SE)	67.09 (1.48)	35.71 (0.74)	5.52 (0.55)	−31.38 (−34.05, −28.71)	−61.57 (−64.18, −58.96)	−30.19 (−31.58, −28.80)
NFS category, percent (SE)	17.01 (1.58)	6.07 (0.34)	1.04 (0.30)	−10.94 (−12.97, −8.91)	−15.97 (−17.97, −13.97)	−5.03 (−5.69, −4.37)

The symbol “†” Indicating: For continuous variables: survey-weighted mean (95% CI), *P*-value was by survey-weighted linear regression (svyglm). For categorical variables: survey-weighted percentage (95% CI), *P*-value was by survey-weighted Chi-square test (svytable).

The symbol “‡” indicates: The effect size for continuous variables is Cohen’s d, prevalence difference for categorical variables. BMI, body mass index; HDL, high-density lipoprotein cholesterol; LDL, low-density lipoprotein cholesterol; HbA1C, glycated hemoglobin; ALT, alanine transaminase; AST, alanine aminotransferase; ALP, alkaline phosphatase; GGT, gamma glutamyltransferase; LE8, life’s essential 8; CVD, cardiovascular and cerebrovascular events; MAFLD, metabolic dysfunction-associated fatty liver disease; NFS, NAFLD fibrosis score.

### 3.2 Association of LE8 with MAFLD or advanced liver fibrosis

Table 2 shows the negative correlation between weighted logistic regression LE8 score and MAFLD in all models. In model 3, OR for per 1 SD increase in LE8 score was 0.64 (95% CI 0.57–0.71) in association with MAFLD. Similarly, fortunately, for every 10 points increase in LE8 score related to MAFLD, the OR was 0.73 (95% CI 0.68–0.79). Compared with the low CVH group, the OR values of MAFLD in the moderate CVH group and high CVH group were 0.66 (95% CI 0.52–0.84) and 0.33 (95% CI 0.23–0.47), respectively; Compared with the low CVH group, there was no significant correlation between the moderate health behaviors group and MAFLD. The OR values of MAFLD in the high CVH group were 0.69 (95% CI 0.53–0.90), respectively. For every standard deviation increase in the health behaviors score, the OR was 0.87 (95% CI 0.87–0.96); Compared with healthy behaviors, health factors are more correlated with MAFLD, with an OR of 0.53 (95% CI 0.46–0.60) for every standard deviation increase in healthy behaviors score. The ORs of MAFLD in the moderate health factors group were 0.67 (95% CI 0.55–0.83) and in the high health factors group were 0.32 (95% CI 0.23–0.43) in comparison to the low health factors group. In sensitivity analysis, participants with a history of CVD history were excluded due to their potential impact on LE8 indicator. The conclusions drawn align with those observed in the fully adjusted model.

**TABLE 2:** Weighted logit regression showing the relationship between LE8/CVH and MAFLD.

	Model 1		Model 2		Model 3		Model 4	
	OR (95% CI)	*p*-value	OR (95% CI)	*p*-value	OR (95% CI)	*p*-value	OR (95% CI)	*p*-value
**LE8 score**								
per 1 SD	0.33 (0.31, 0.35)	<0.001	0.30 (0.28, 0.32)	<0.0001	0.64 (0.57, 0.71)	<0.001	0.61 (0.54, 0.69)	<0.001
Per 10 points increase	0.46 (0.45, 0.48)	<0.001	0.43 (0.41, 0.45)	<0.0001	0.73 (0.68, 0.79)	<0.001	0.71 (0.65, 0.77)	<0.001
Low CVH (0–49)	Ref.		Ref.		Ref.		Ref.	
Moderate CVH (50–79)	0.27 (0.24, 0.31)	<0.001	0.24 (0.20, 0.28)	<0.0001	0.66 (0.52, 0.84)	0.001	0.59 (0.45, 0.77)	0.003
High CVH (80–100)	0.03 (0.02, 0.04)	<0.001	0.03 (0.02, 0.04)	<0.0001	0.33 (0.23, 0.47)	<0.001	0.30 (0.20, 0.45)	<0.001
**Health behaviors score**								
per 1 SD	0.80 (0.76, 0.84)	<0.001	0.80 (0.75, 0.85)	<0.0001	0.87 (0.78, 0.96)	0.007	0.85 (0.76, 0.96)	0.007
Per 10 points increase	0.89 (0.87, 0.91)	<0.001	0.89 (0.87, 0.92)	<0.0001	0.93 (0.88, 0.98)	0.007	0.92 (0.87, 0.98)	0.007
Low CVH (0–49)	Ref.		Ref.		Ref.		Ref.	
Moderate CVH (50–79)	0.92 (0.82, 1.04)	0.170	0.92 (0.81, 1.05)	0.2062	0.87 (0.69, 1.09)	0.231	0.84 (0.65, 1.08)	0.185
High CVH (80–100)	0.53 (0.45, 0.61)	<0.001	0.54 (0.45, 0.64)	<0.0001	0.69 (0.53, 0.90)	0.007	0.68 (0.50, 0.91)	0.013
**Health factors score**								
per 1 SD	0.21 (0.19, 0.22)	<0.001	0.17 (0.16, 0.19)	<0.0001	0.53 (0.46, 0.60)	<0.001	0.49 (0.42, 0.57)	<0.001
per10 points increase	0.44 (0.42, 0.45)	<0.001	0.40 (0.38, 0.41)	<0.0001	0.71 (0.66, 0.77)	<0.001	0.69 (0.64, 0.75)	<0.001
Low CVH (0–49)	Ref.		Ref.		Ref.		Ref.	
Moderate CVH (50–79)	0.22 (0.19, 0.25)	<0.001	0.18 (0.16, 0.21)	<0.0001	0.67 (0.55, 0.83)	<0.001	0.68 (0.53, 0.86)	0.002
High CVH (80–100)	0.02 (0.02, 0.02)	<0.001	0.02 (0.01, 0.02)	<0.0001	0.32 (0.23, 0.43)	<0.001	0.30 (0.21, 0.43)	<0.001

OR odds ratio, CI confidence interval, LE8, life’s essential 8; CVH, cardiovascular health; Low CVH was defined as a LE8 score of 0 to 49, moderate CVH of 50–79, and high CVH of 80–100;

Model 1: Unadjusted model;

Model 2: Adjusted for age (as a continuous variable), sex, race, marital status, Education levels, poverty-income ratio.

Model 3: Additionally adjusted for BMI (< 25, 25–30, ≥ 30), GGT, HDL, TG, ALT, AST, ALP, alcohol, albumin, smoking, hypertension, CVD, diabetes, depression, and cancer.

Model 4: Excluding CVD history participants; adjusted for age (as a continuous variable), sex, race, marital status, Education levels, poverty-income ratio, BMI (< 25, 25–30, ≥ 30), GGT, HDL, TG, ALT, AST, ALP, alcohol, albumin, smoking, hypertension, diabetes, depression, and cancer.

For liver fibrosis, the association between LE8 and health behaviors/factors with advanced liver fibrosis was documented in Table 3. In model 3, there was no significant association observed between the moderate and high health behaviors group and advanced liver fibrosis, even when compared to the low CVH group. For per 1 SD increase in LE8 score, the probability of advanced liver fibrosis among participants decreases by 25% (OR 0.75, 95% CI 0.61–0.9); The OR for every 10 points increase in the LE8 score is 0.82 (95% CI 0.71–0.94). We found that there was no significant correlation between healthy behaviors components and advanced liver fibrosis. Compared to the low health factors group, the OR for advanced liver fibrosis was 0.62 (95% CI 0.46–0.83) in the moderate health factors group and 0.44 (95% CI 0.23–0.83) in the high health factors group. Furthermore, for every 10-point increase in health factors, the OR for advanced liver fibrosis was 0.78 (95% CI 0.69–0.87). In sensitivity analysis, we observed that the association between MAFLD, advanced liver fibrosis, and LE8/health factors remained robust. No significant correlation was detected between components of healthy behaviors and advanced liver fibrosis.

**TABLE 3:** Weighted logit regression showing the relationship between LE8/CVH and advanced liver fibrosis.

	Model 1		Model 2		Model 3		Model 4	
	**OR (95% CI)**	***p***	**OR (95% CI)**	***p***	**OR (95% CI)**	***p***	**OR (95% CI)**	***p***
**LE8 score**								
per 1 SD	0.42 (0.37, 0.47)	<0.001	0.44 (0.38, 0.50)	<0.001	0.75 (0.61, 0.92)	0.007	0.75 (0.58, 0.99)	0.043
per10 points increase	0.54 (0.50, 0.59)	<0.001	0.56 (0.51, 0.62)	<0.001	0.82 (0.71, 0.94)	0.007	0.82 (0.68, 0.99)	0.043
Low CVH (0–49)	Ref.		Ref.		Ref.		Ref.	
Moderate CVH (50–79)	0.32 (0.24, 0.41)	<0.001	0.34 (0.25, 0.45)	<0.001	0.75 (0.51, 1.10)	0.145	0.85 (0.52, 1.40)	0.532
High CVH (80–100)	0.05 (0.03, 0.10)	<0.001	0.09 (0.04, 0.17)	<0.001	0.68 (0.32, 1.44)	0.322	0.75 (0.29, 1.93)	0.546
**Health behaviors score**								
per 1 SD	0.85 (0.76, 0.94)	0.003	0.84 (0.74, 0.94)	0.004	0.93 (0.78, 1.10)	0.407	0.95 (0.77, 1.18)	0.655
per10 points increase	0.92 (0.87, 0.97)	0.003	0.91 (0.86, 0.97)	0.004	0.96 (0.88, 1.05)	0.407	0.98 (0.88, 1.09)	0.655
Low CVH (0–49)	Ref.		Ref.		Ref.		Ref.	
Moderate CVH (50–79)	0.72 (0.58, 0.90)	0.005	0.74 (0.57, 0.95)	0.020	0.77 (0.55, 1.07)	0.126	0.84 (0.55, 1.26)	0.398
High CVH (80–100)	0.59 (0.43, 0.79)	0.001	0.59 (0.43, 0.81)	0.002	0.85 (0.57, 1.27)	0.440	0.99 (0.60, 1.62)	0.961
**Health factors score**								
Per 1 SD	0.29 (0.26, 0.33)	<0.001	0.33 (0.29, 0.37)	<0.001	0.62 (0.50, 0.77)	<0.001	0.61 (0.45, 0.81)	0.001
Per10 points increase	0.52 (0.49, 0.55)	<0.001	0.56 (0.52, 0.60)	<0.001	0.78 (0.69, 0.87)	<0.001	0.77 (0.66, 0.90)	0.001
Low CVH (0–49)	Ref.		Ref.		Ref.		Ref.	
Moderate CVH (50–79)	0.22 (0.17, 0.27)	<0.001	0.24 (0.19, 0.30)	<0.001	0.62 (0.46, 0.83)	0.002	0.57 (0.40, 0.82)	0.003
High CVH (80–100)	0.03 (0.02, 0.04)	<0.001	0.06 (0.04, 0.10)	<0.001	0.44 (0.23, 0.83)	0.013	0.50 (0.24, 1.06)	0.074

OR odds ratio, CI confidence interval, LE8, life’s essential 8; CVH, cardiovascular health;

Low CVH was defined as a LE8 score of 0 to 49, moderate CVH of 50–79, and high CVH of 80–100;

Model 1: Unadjusted model;

Model 2: Adjusted for age (as a continuous variable), sex, race, marital status, Education levels, poverty-income ratio.

Model 3: Additionally adjusted for BMI (< 25, 25–30, ≥ 30), GGT, HDL, TG, ALT, AST, ALP, alcohol, albumin, smoking, hypertension, CVD, diabetes, depression, and cancer.

Model 4: Excluding CVD history participants; adjusted for age (as a continuous variable), sex, race, marital status, education levels, poverty-income ratio, BMI (< 25, 25–30, ≥ 30), GGT, HDL, TG, ALT, AST, ALP, alcohol, albumin, smoking, hypertension, diabetes, depression, and cancer.

### 3.3 Smooth curve fitting

**FIGURE 1:**
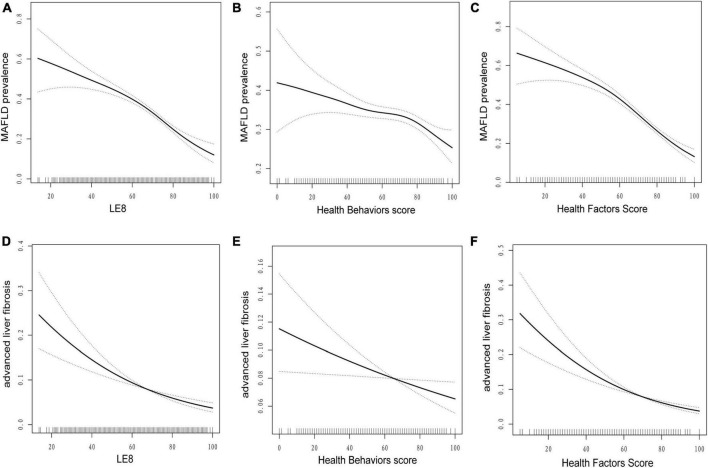
Dose–response relationships between life’s essential 8 scores **(A,D)**, health behavior score **(B,E)**, health factors score **(C,F)**, and metabolic dysfunction-associated fatty liver disease (MAFLD). Model adjusted for age (as a continuous variable), sex, race, marital status, education levels, poverty-income ratio, BMI (< 25, 25–30, ≥ 30), GGT, HDL, TG, ALT, AST, ALP, alcohol, albumin, smoking, hypertension, CVD, diabetes, depression, and cancer. LE8, life’s essential 8; MAFLD, metabolic dysfunction-associated fatty liver disease.

Figure 1 shows a smooth curve fitting graph based on the generalized additive model to visualize the association between LE8 and its subscales score and incidence of MAFLD and advanced liver fibrosis. Figures 1A–C illustrate a non-linear negative association between LE8 score, health behaviors/factors and MAFLD. Figures 1D–F shows advanced liver fibrosis decreases with the increase of LE8 and health behaviors/factors, respectively.

### 3.4 Subgroup analysis

**FIGURE 2:**
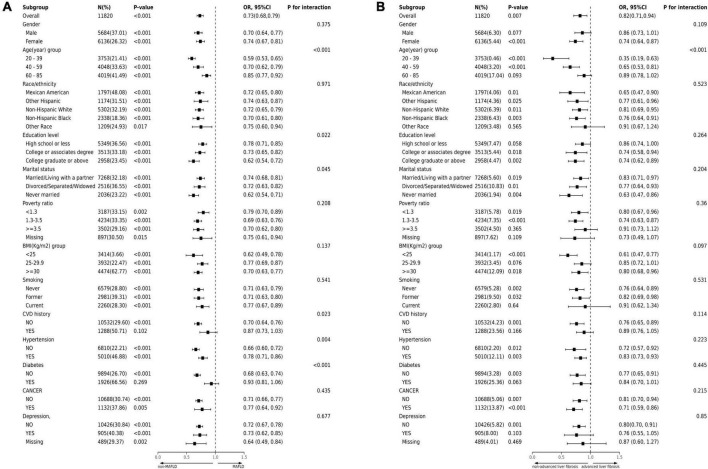
Subgroup analysis of the association of CVH metrics with **(A)** MAFLD and **(B)** advanced liver fibrosis. The results were adjusted for all covariates except for the corresponding stratification variable. CVH, cardiovascular health; MAFLD, metabolic dysfunction-associated fatty liver disease.

We employed stratified weighted multivariate regression analysis to investigate the association between MAFLD, advanced liver fibrosis, and LE8 score within a population stratified by sex, age, race, education level, marital status, PIR, smoking status, BMI, hypertension, diabetes, cancer, CVD, and depression. Higher LE8 score were consistently associated with a decreased incidence of MAFLD across nearly all stratified subgroups. However, the association between LE8 score and incidence of MAFLD was not statistically significant in participants who were CVD history or diabetes (*P* > 0.05) (Figure 2A). The interaction test showed that gender, race, PIR, smoking, cancer, and depression had no significant impact on the association between LE8 score and MAFLD (all *P* for interaction > 0.05). However, age, education level, marital status, CVD, hypertension and diabetes significantly influenced this association (interaction *p* < 0.05). Additionally, within the majority of subgroups, a significant inverse association was observed between advanced liver fibrosis and LE8 score. Notably, there is a significant interaction between the LE8 score and age in advanced liver fibrosis patients (*P* < 0.05). Among male, elderly, wealthy, other ethnic, CVD, diabetes and depression participants, the correlation between LE8 score and advanced liver fibrosis was not statistically significant (*P* > 0.05) (Figure 2B).

## 4 Discussion

This study conducted among participants in the US NHANES (2005–2018) has affirmed our hypothesis that adults with higher levels of cardiovascular health (CVH) metrics assessed by LE8 score have a reduced risk of MAFLD and advanced liver fibrosis. We observed a nonlinear dose-response relationship between increased LE8 score and decreased risk of MAFLD and advanced liver fibrosis. For per 10-point increase in LE8 scores, the risk of MAFLD decreased by 27% and the risk of advanced liver fibrosis decreased by 18%. As LE8 is the latest improvement in evaluating cardiovascular health (CVH), this study enhances the association between cardiovascular health and MAFLD and advanced liver fibrosis. Improving the LE8 score may offer clinical benefits as a feasible and effective means to promote liver wellbeing.

Our findings support previous studies that participants with ideal CVH metrics tend to have a lower risk of developing NAFLD. In Multi Ethnic Study of Atherosclerosis (MESA) cohort, a more feasible LS7 level was associated with a lower priority of NAFLD (34). In Jang’s study, regression of already existing NAFLD and a lower incidence of NAFLD were both significantly correlated with higher CVH metrics (35). Wang et al. (21) discovered that the higher the LE8 score was associated with a lower prevalence of NAFLD (21). A cross-sectional study conducted in Northern China found a correlation between NAFLD prevalence and quartiles of cardiovascular health summary scores. Individuals in the highest quartile had reduced odds (OR) compared to those in the lowest quartile (adjusted OR: 0.17, 95% CI: 0.17–0.20). This association remained consistent across different gender and age groups (36). Compared to other research on the association between CVH metrics and NAFLD, our study further emphasizes the liver’s role in relation to CVH. Our study incorporates a wide range of liver-related covariates, metabolic factors, and chronic non-communicable diseases, thereby enhancing the robustness and reliability of our findings. Moreover, our results advocate for increased emphasis on CVH metrics within the general population of the United States, aiming to mitigate the risk of cardiovascular disease, as well as the incidence of MAFLD and advanced liver fibrosis (20).

Sleep disorders are related to the pathogenesis of chronic liver disease, especially the occurrence and progression of non-alcoholic fatty liver disease. Due to the lack of evaluation of sleep by LS7, the body’s healthy characteristics and behaviors may not be entirely reflected in the definition of LS7 (37). And the insensitivity of the CHV definition of LS7 to individual differences, it cannot be used to evaluate dose-response effects. This study provided significant demonstration of the connection between CVH and MAFLD, advanced liver fibrosis by applying LE8 score as the definition of CVH metrics. We found that in the health factor scores related to MAFLD, these findings indicate that LE8 score improve the quantification method of CVH metrics and increase the sensitivity of scores to individual and group differences (12). Participants with higher levels of ideal behavioral and metabolic factors have a lower risk of MAFLD and advanced liver fibrosis. These findings additionally highlight the variations in the potential benefits of CHV metrics, and the promotion of CVH requires the carrying out of a population-level strategy.

Elevated LE8 scores may contribute to a reduction in the incidence of MAFLD through the enhancement of health behaviors and factors. Research has shown that MAFLD is a manifestation of metabolic syndrome and insulin resistance in the liver, which are intrinsic health factors and health behaviors indicators of LE8 (38). Emerging research has explored the role of certain components of CVH metrics in the incidence of MAFLD and liver fibrosis. Altaf et al. (39) proposed that implementing a healthy lifestyle via exercise led to a decrease in BMI, objective measurements, enhanced glycomic regulation, and a reversal of the live fat content with better live enzymes in the MAFLD group. Physical activity speeds up the body’s metabolism of fat and glucose, decreases the production of inflammatory markers, and increases the sensitivity of the liver and skeletal muscles to the insulin response (40, 41). Yang et al. (42) reported that sleeping late, snoring, and taking a nap for more than 30 min during the day were significantly associated with an increased risk of MAFLD, with participants with nighttime sleep disorders and prolonged daytime naps having the highest risk of MAFLD (OR: 2.38, 95% CI: 1.73–3.27) (42). Smoking is related to metabolism and may also worsen MAFLD by enhancing pro-inflammatory cytokines and oxidative stress (43). Inflammation also has a significant impact on CVD and MAFLD. According to reports, MAFLD could cover more FLD than NAFLD, and the MAFLD-only group had a more severe inflammation status (44). The association between obesity and MAFLD, typically characterized by low-grade inflammation, represents a chronic metabolic disorder. The Previous research has demonstrated that an Eight Week Very Low-Calorie Ketogenic Diet (VLCKD) effectively reduces white blood cell (WBC) and platelet (PLT) counts, and exhibits efficacy in ameliorating hepatic steatosis and fibrosis. Thus, altering dietary behavior and structure presents a feasible strategy for preventing and managing MASLD (45). Xiong et al. (46) reported that inflammation is an important mechanism regulating body metabolism, which in turn affects chronic metabolic diseases. It affects systemic regulation of metabolism through a complex multi-organ crosstalk network including several signaling pathways such as NLRP3/caspase-1/IL-1, NF-B, p38 MAPK, IL-6/STAT3, and PI3K/AKT (46). The increase in circulating inflammatory markers is also related to MAFLD. Impaired glycemic control and systemic insulin resistance may promote increased flux of free fatty acids from peripheral tissues to the liver, thereby predisposing to the development and progression of non-alcoholic fatty liver disease (NAFLD) even before the onset of diabetes mellitus (47). Ajmera et al. (48) received that type 2 diabetic individuals have greater rates of liver fibrosis and steatosis (48). In a US study, HbA1c was significantly associated with liver steatosis and fibrosis. Strengthening glycomic regulation could possibly affect the likelihood of NASH-related fibrosis advancement (49). Research has shown that Remnant cholesterol was independently associated with the risk of MAFLD (50). In recent years, the research and development of drugs in the treatment of MAFLD has made rapid progress. Semaglutide, a glucagon-like peptide-1 (GLP-1) receptor agonist, has demonstrated efficacy in improving clinical indices of liver enzymes and reducing hepatic steatosis. They can induce significant weight loss and increase insulin sensitivity. In particular, they can reduce de novo lipogenesis, enhance mitochondrial β-oxidation of free fatty acids, decrease systemic and liver insulin resistance, and increase the clearance of very low-density lipoproteins. These effects suggest that semaglutide represents a promising therapeutic strategy for the medical treatment of MAFLD (51, 52). Many previous studies have focused on individual factors related to NAFLD. The LE8 score represents a comprehensive and user-friendly assessment tool within clinical settings, promoting adherence to optimal health factors and healthy behaviors in the field of biomedical science. Our research expands the scope of healthy behaviors and ideal health factors. In conclusion, it is not surprising that there is a significant association between LE8 score, health factors/behaviors and the incidence of MAFLD and advanced liver fibrosis.

The primary strength of this study lies in the utilization of a large, nationally representative sample of American adults, facilitating the generalizability of the research findings to a broader population within the biomedical field. In addition, we discussed the dose-response relationship between MAFLD, advanced liver fibrosis and each component of the CVH metrics. This study still has some limitations. The primary limitation of our study lies in utilizing non-invasive USFLI and NFS scores as diagnostic criteria for hepatic steatosis and advanced liver fibrosis. Although they have been validated as reliable for diagnosing hepatic steatosis and fibrosis in the American population (26), it must be acknowledged that histological diagnosis remains the gold standard. Therefore, when employing these non-invasive scoring methods, there is a possibility of inaccuracies in estimating the true risk magnitude of MAFLD and advanced liver fibrosis due to incorrect classification or potential underestimation or overestimation of disease prevalence. Secondly, considering the inherent limitations of the cross-sectional design of this study, establishing a causal relationship between LE8 and MAFLD, as well as advanced liver fibrosis, is not feasible. Therefore, it is imperative for a well-designed prospective study to further investigate the impact of LE8 on the incidence of MAFLD and advanced liver fibrosis, and for our observations to be validated. Finally, the evaluation of health behaviors indicators is based on self-reported questionnaires, which may have measurement errors.

## 5 Conclusion

In this nationally representative sample of US adults, the ideal CVH metrics may be beneficial to significantly reduce the risk of liver steatosis and fibrosis. The findings of our study indicate a potential beneficial role of LE8 as a practical and effective approach to reduce the burden of MAFLD and advanced liver fibrosis. Furthermore, our findings may raise awareness among the general population about the importance of living a healthy lifestyle.

## Data availability statement

The datasets presented in this study can be found in online repositories. The names of the repository/repositories and accession number(s) can be found in this article/Supplementary material.

## Ethics statement

The studies involving humans were approved by the National Center for Health Statistics Ethics Review Committee, and informed consent was acquired via documentation from each participant (https://www.cdc.gov/nchs/data_access/restrictions.htm). The studies were conducted in accordance with the local legislation and institutional requirements. The participants provided their written informed consent to participate in this study. Written informed consent was obtained from the individual(s) for the publication of any potentially identifiable images or data included in this article.

## Author contributions

DL: Conceptualization, Methodology, Supervision, Visualization, Writing – original draft, Writing – review & editing. JZ: Conceptualization, Data curation, Writing – original draft, Writing – review & editing. LL: Conceptualization, Validation, Writing – review & editing. YL: Conceptualization, Validation, Writing – original draft. LX: Conceptualization, Funding acquisition, Supervision, Validation, Writing – original draft, Writing – review & editing. HW: Conceptualization, Funding acquisition, Supervision, Validation, Writing – original draft, Writing – review & editing.

## Footnotes

1
https://www.cdc.gov/nchs/data_access/restrictions.htm

2
www.empowerstats.com
