# Supplementary material for: Associations of life’s essential 8 with MAFLD and liver fibrosis among US adults: a nationwide cross-section study
Source: Front Nutr. 2024 Jun 12;11:1403720. doi: 10.3389/fnut.2024.1403720 (PMC11199778; doi:10.3389/fnut.2024.1403720)
Supplement: Supplementary file 1 [file Data_Sheet_1.pdf]

# *Supplementary Material*

## 1 Supplementary Figure 1

Flow chart of the screening process for the selection of the study population

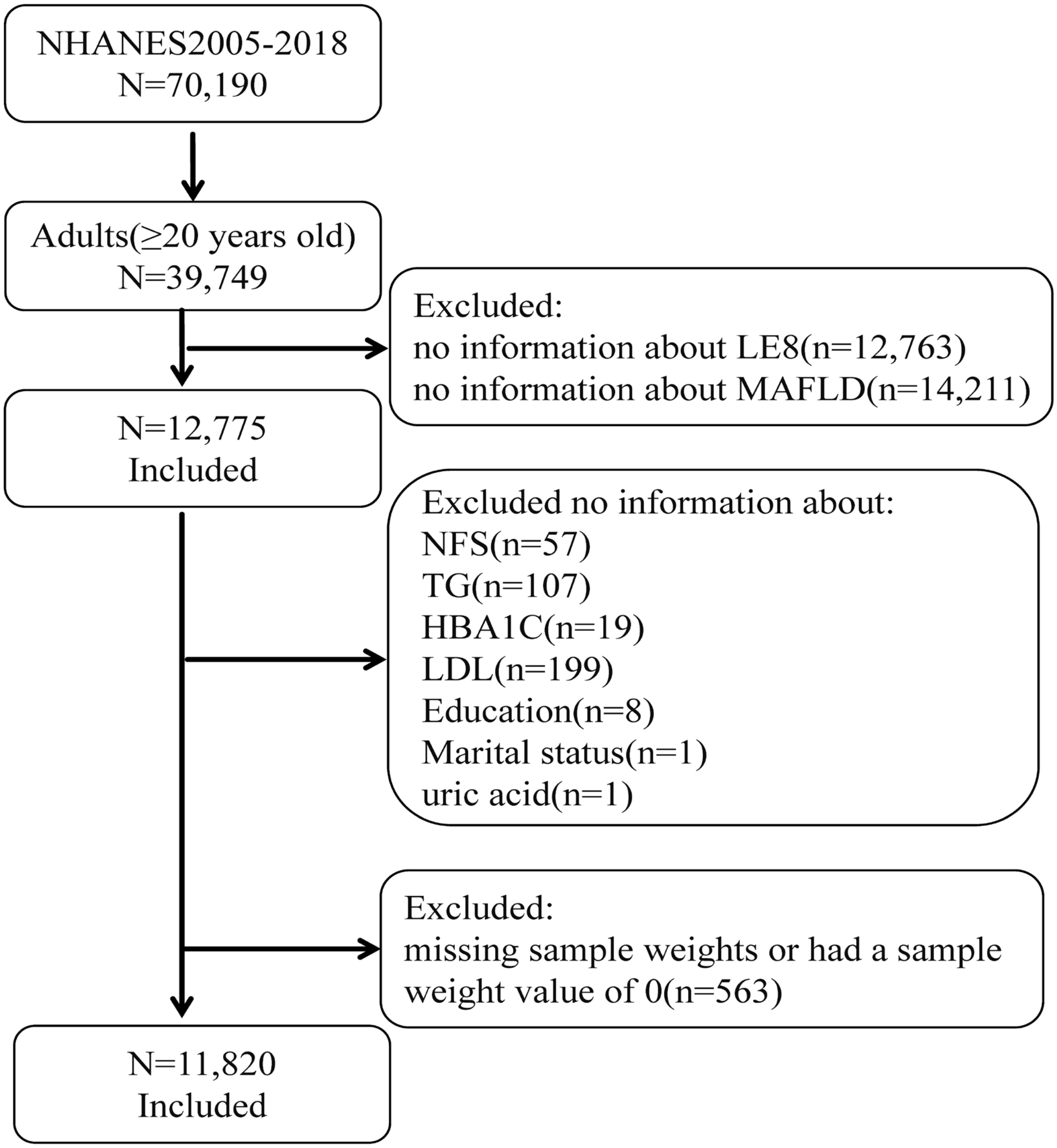


Abbreviations: NHANES: National Health and Nutrition Examination Survey; LE8: Life's Essential 8; MAFLD: metabolic dysfunction-associated fatty liver disease; LDL: low-density lipoprotein cholesterol; HbA1C: glycated hemoglobin; TG: Triglycerides; NFS: NAFLD fibrosis score.

**2. Supplementary Table S1**

**VIF test**

| | Step 1 |
|---|---|
| LE8 | 2.5 |
| GENDER | 1.4 |
| AGE | 1.7 |
| Race/ethnicity | 1.2 |
| Marital status | 1.1 |
| Education levels | 1.3 |
| PIR | 1.2 |
| HDL | 1.6 |
| TG | 1.4 |
| ALT | 3.1 |
| AST | 2.7 |
| SMOKING | 1.5 |
| CVD | 1.2 |
| HBP | 1.5 |
| T2DM | 1.3 |
| CANCER | 1.1 |
| BMI | 1.6 |

| | |
|---|---|
| GGT | 1.4 |
| ALP | 1.2 |
| DEPRESSION | 1 |
| ALCOHOL | 1.2 |
| ALBUMINSI | 1.3 |

3. **Supplementary Table S2**

Definition and scoring approach for the American Heart Association's Life's Essential 8 score.

| Domain | CVH Metric | Measurement | Quantification and Scoring of CVH Metric : adults(≥ 20 years of age) |
|---|---|---|---|
| Health Behaviors | Diet | Healthy Eating Index-2015 diet score percentile | Quantiles of DASH-style diet adherence<br>**Scoring (Population):**<br>Points Quantile<br>100 ≥95th percentile (top/ideal diet)<br>80 75th – 94th percentile<br>50 50th – 74th percentile<br>25 25th – 49th percentile<br>0 1st – 24th percentile (bottom/least ideal quartile) |
| | Physical activity | Self-reported minutes of moderate or vigorous physical activity per week | **Metric:** Minutes of moderate (or greater) intensity activity per week<br>**Scoring:**<br>Points Minutes<br>100 ≥150<br>90 120 – 149<br>80 90 – 119<br>60 60 – 89<br>40 30 – 59<br>20 1 – 29<br>0 0 |

| | | | |
|---|---|---|---|
| | Nicotine exposure | Self-reported use of cigarettes or inhaled nicotine- delivery system | **Metric:** Combustible tobacco use and/or inhaled NDS use; or secondhand smoke exposure<br>**Scoring:**<br>Points Status<br>100 Never smoker<br>75 Former smoker, quit ≥5 yrs<br>50 Former smoker, quit 1 - <5 yrs<br>25 Former smoker, quit <1 year, or currently using inhaled NDS<br>0 Current smoker<br>Subtract 20 points (unless score is 0) for living with active indoor smoker in home |
| | Sleep health | Self-reported average hours of sleep per night | **Metric:** Average hours of sleep per nigh<br>**Scoring:**<br>Points Level<br>100 7 – <9<br>90 9 – <10<br>70 6 – <7<br>40 5 – <6 or ≥10<br>20 4 – <5<br>0 <4 |
| Health Factors | Body mass index | Body weight (kg) divided by height squared ($m^2$) | **Metric:** Body mass index ($kg/m^2$)<br>**Scoring:** Points<br>Level 100<br><25<br>70 25.0 – 29.9<br>30 30.0 – 34.9<br>15 35.0 – 39.9<br>0 ≥40.0 |
| | Blood lipids | Plasma total and HDL-cholesterol with calculation of non-HDL-cholesterol | **Metric:** Non-HDL-cholesterol (mg/dL)<br>**Scoring:**<br>Points Level<br>100 <130<br>60 130 – 159<br>40 160 – 189<br>20 190 – 219<br>0 ≥220<br>If drug-treated level, subtract 20 points |
| | Blood glucose | Fasting blood glucose or casual hemoglobin A1c | **Metric:** Fasting blood glucose (mg/dL) or Hemoglobin A1c (%)<br>**Scoring:**<br>Points Level |

| | | | |
|---|---|---|---|
| | | | 100 No history of diabetes and FBG <100 (or HbA1c < 5.7)<br>60 No diabetes and FBG 100 – 125 (or HbA1c 5.7-6.4) (Pre-diabetes)<br>40 Diabetes with HbA1c <7.0<br>30 Diabetes with HbA1c 7.0 – 7.9<br>20 Diabetes with HbA1c 8.0 – 8.9<br>10 Diabetes with Hb A1c 9.0 – 9.9<br>0 Diabetes with HbA1c ≥10.0 |
| | Blood pressure | Appropriately measured systolic and diastolic blood pressure | **Metric:** Systolic and diastolic blood pressure (mmHg)<br><br>**Scoring:**<br><u>Points Level</u><br>100 <120/<80 (Optimal)<br>75 120-129/<80 (Elevated)<br>50 130-139 or 80-89 (Stage I HTN)<br>25 140-159 or 90-99<br>0 ≥160 or ≥100<br><br>Subtract 20 points if treated level |
